# Summary of the National Advisory Committee on Immunization (NACI) Statement: Updated recommendations on herpes zoster vaccination for adults who are immunocompromised

**DOI:** 10.14745/ccdr.v52i0102a01

**Published:** 2026-02-19

**Authors:** Ramya Krishnan, Oliver Baclic, Ana Howarth, Ashleigh Tuite, Melissa Andrew

**Affiliations:** 1 Centre for Immunization Surveillance and Programs, Public Health Agency of Canada, Ottawa, ON; 2 Department of Medicine, Division of Geriatric Medicine, Dalhousie University, Halifax, NS

**Keywords:** National Advisory Committee on Immunization, herpes zoster, shingles, Canada, immunocompromised, Shingrix, recombinant zoster vaccine, vaccine guidance

## Abstract

**Background:**

Herpes zoster (HZ), or shingles, results from the reactivation of latent varicella-zoster virus and poses a significant health burden and immunocompromised adults are at higher risk of HZ and its complications. In 2018, the recombinant zoster vaccine (RZV, Shingrix®) was strongly recommended by the National Advisory Committee on Immunization (NACI) for immunocompetent adults aged 50 years and older. Since then, evidence has accumulated on the use of RZV in immunocompromised adults and in 2021, Health Canada expanded the authorization of RZV to adults 18 years of age and older who are or will be immunocompromised.

**Methods:**

NACI assessed the burden of HZ in immunocompromised populations, reviewed evidence on the efficacy, effectiveness, immunogenicity and safety of RZV, and published economic evaluations. Programmatic considerations were evaluated using NACI’s ethics, equity, feasibility and acceptability framework. The evidence and programmatic considerations were organized using a process informed by the *Grading of Recommendations, Assessment, Development and Evaluation* framework, and this information was then used to facilitate NACI guidance development.

**Results:**

The risk of HZ among younger adults who are immunocompromised is comparable to or higher than the general population of 50 years of age and older. High efficacy and robust immune responses after RZV administration was demonstrated in groups with various types of immunocompromising therapies and conditions, with an acceptable safety profile. Economic evaluations showed that RZV was cost-effective in some high-risk immunocompromised groups. Expanding access to RZV may reduce disease burden and address inequities in vaccine access.

**Conclusion:**

NACI updated its guidance to strongly recommend that individuals 18 years of age and older who are or will be immunocompromised should receive two doses of RZV to prevent HZ and its associated complications.

## Introduction

Herpes zoster (HZ), commonly known as shingles, poses a substantial public health burden in Canada and globally. Herpes zoster is caused by a reactivation of varicella-zoster virus (VZV) and typically presents as a painful, unilateral vesicular rash usually localized to one dermatome, though it can result in a range of complications requiring medical intervention, such as postherpetic neuralgia (PHN), which can lead to chronic disabling pain. While anyone who has had varicella is at risk of developing HZ, it occurs more frequently among older adults and persons who are immunocompromised ((1,2)).

The National Advisory Committee on Immunization (NACI) last issued guidance on HZ vaccination in 2018, recommending that the recombinant zoster vaccine (RZV, Shingrix®) should be offered to immunocompetent adults 50 years of age and older (strong NACI recommendation) and may be considered for adults 50 years of age and older who are immunocompromised (discretionary NACI recommendation) ((3)). NACI was asked to review public health recommendations following Heath Canada’s authorization on November 24, 2021 ((4)), of RZV for adults 18 years of age or older who are or will be at increased risk of HZ due to immunodeficiency or immunosuppression caused by known disease or therapy.

## Methods

NACI reviewed evidence on the burden of disease for HZ and HZ-related complications in Canada, evidence on RZV efficacy, effectiveness, immunogenicity and safety in adults who are immunocompromised, and published economic evaluations comparing RZV to no vaccine in adults who are immunocompromised ((5,6)). NACI used a published, peer-reviewed framework and evidence-informed tools to ensure that issues related to ethics, equity, feasibility and acceptability were systematically assessed and integrated into the guidance. NACI also considered feedback from the Canadian Immunization Committee and leveraged a 2022 evidence synthesis from the United States (US) Centers for Disease Control and Prevention and Advisory Committee on Immunization Practices, which included a *Grading of Recommendations, Assessment, Development and Evaluation* (GRADE) assessment and an Evidence to Recommendations framework on RZV vaccine efficacy, effectiveness, immunogenicity and safety among individuals aged 19 years and older who are or will be at increased risk of HZ due to immunodeficiency or immunosuppression caused by known disease or therapy ((7–9)). Knowledge synthesis was performed by the NACI secretariat and reviewed by the Herpes Zoster Working Group. For complete details of the methods, refer to the NACI statement ((10)).

## Results

### Burden of disease in Canada

In Canada, the lifetime risk of developing HZ is estimated to be as high as 30% in the general population, with incidence and severity increasing markedly in older adults and in those with compromised immunity. A model has estimated that each year, approximately 130,000 new cases of HZ occur nationally, leading to about 17,000 cases of PHN, 252,000 physician consultations and 2,000 hospitalizations ((11)). A systematic review of Canadian data found that incidence rates of medically-attended HZ ranged between 316 and 450 per 100,000 person-years across several provinces ((12)).

Individuals who are immunocompromised face particularly elevated risk. An Ontario-based study reported that adults with immunocompromising conditions were 2.9 to 12.3 times more likely to experience hospital-attended HZ compared to immunocompetent individuals, after adjusting for age and sex ((13)). The incidence rates of HZ were higher for younger adults with immunosuppression and were similar to or greater than those of immunocompetent older adults. Similar findings from the US have shown HZ incidence rates of 17 to 43 per 1,000 person-years in specific immunocompromised groups (e.g., adults with solid organ transplant, adults with bone marrow or stem cell transplant and adults with HIV), compared to 4.8 per 1,000 person-years in the general adult population ((14)). Herpes zoster risk is lower for individuals who have only been exposed to VZV through vaccination with live-attenuated virus compared to those who have been exposed through infection ((15–17)).

The public health importance of the burden of disease due to HZ is underscored by the prevalence of individuals in Canada who are immunocompromised and therefore at high risk of HZ and its associated complications. Over 155,000 Canadians are estimated to be living with a hematologic malignancy, more than 62,000 are living with HIV and approximately 29,000 have primary immunodeficiencies ((18–20)). Between 2000 and 2019, more than 18,000 first hematopoietic stem cell transplants were performed in Canada ((21)).

Complications from acute HZ are more common in these populations, and potentially more severe. A systematic review reported that the risk of developing PHN ranged between 6% and 45% across immunocompromising conditions ((22)). This is particularly important given that PHN can impair quality of life to a degree comparable to serious chronic illnesses, including diabetes, myocardial infarction, congestive heart failure and depression ((23)).

### Vaccine efficacy and effectiveness

Evidence from randomized-controlled trials (RCTs) demonstrated that a two-dose schedule of RZV is efficacious in preventing HZ in adults with a range of immunocompromising conditions. In RCTs, vaccine efficacy was 68% (95% CI: 56%–78%) in autologous hematopoietic stem cell transplant (HSCT) recipients and 87% (95% CI: 44%–99%) and 90.5% (95% CI: 74%–98%) in individuals with hematologic malignancies and immune-mediated diseases, respectively ((7,9)). Observational studies supported these findings, with vaccine effectiveness estimates of 64% (95% CI: 57%–70%) and 68% (95% CI: 62%–73%) for individuals with immunocompromising conditions and autoimmune conditions, respectively ((7)). Additionally, RZV was efficacious for the prevention of HZ-related hospitalization and PHN in HSCT recipients ((7)).

### Vaccine immunogenicity

Recombinant zoster vaccine was demonstrated to be immunogenic in RCTs across a broad range of immunocompromising conditions, including autologous HSCT recipients, individuals with hematological malignancies, individuals with solid tumours, renal transplant recipients and people living with HIV ((7)). One study also showed that RZV immunogenicity was not impaired in individuals with autoimmune conditions treated with immune-targeted therapies when compared to individuals who were not treated with immunosuppressive therapies ((24)).

Immunogenicity was also assessed in RCTs where RZV was administered concurrently with or separately from one other vaccine—either quadrivalent inactivated influenza vaccine, 23-valent pneumococcal polysaccharide vaccine, COVID-19 mRNA-1273, reduced-antigen-content diphtheria-tetanus-acellular pertussis vaccine, 13-valent pneumococcal conjugate vaccine, or respiratory syncytial virus prefusion F3 subunit vaccine for older adults ((25–30)). Immune responses were comparable between concurrent and sequential administration. All immune responses met pre-specified non-inferiority criteria when comparing concurrent administration to sequential administration, except for one of the pertussis antigens in the tetanus, diphtheria and pertussis vaccine; study authors concluded that there is likely no clinically relevant interference between RZV and tetanus, diphtheria and pertussis vaccine.

### Vaccine safety

Recombinant zoster vaccine has an acceptable safety profile that is comparable between immunocompromised and immunocompetent populations. In clinical trials conducted in immunocompromised populations, rates of serious events, risk of immune-mediated diseases, risk of graft-versus-host disease and risk of graft rejection were similar between vaccine and placebo recipients ((7)). The frequency of Grade 3 local and systemic reactogenicity was higher among vaccine recipients compared to placebo recipients ((7)), with pain at the injection site, fatigue and myalgia being the most commonly reported reactions. In studies assessing concurrent administration of RZV with one other vaccine (described above), no safety concerns were identified, with similar frequencies of local and systemic reactogenicity between sequential and concurrent administration groups.

### Economic considerations

Two US-based economic evaluations were reviewed to assess the cost-effectiveness of RZV in immunocompromised adults aged under 50 years ((5,6)). Both studies found that RZV was cost-effective for certain high-risk groups such as HSCT recipients, individuals with multiple myeloma and renal transplant recipients. For other risk groups, incremental cost-effectiveness ratios ranged from $47,900 to $296,360 CAD per quality-adjusted life year gained, depending on the specific condition, model structure and perspective (healthcare sector vs. societal) ((5,6)). In these economic evaluations, the cost-effectiveness of RZV was strongly influenced by the incidence and healthcare costs of HZ, which vary considerably among immunocompromised adults, especially those with autoimmune and inflammatory conditions. Notably, the vaccine prices used in these economic evaluations ($271 CAD and $233 CAD per dose) ((5,6)) were higher than the current Canadian list price for RZV ($162.35 CAD per dose). While these analyses were conducted in the US context, this difference in vaccine prices indicates that, all else being equal, cost-effectiveness estimates would be more favourable if Canadian price assumptions were applied. Although these economic evaluations were conducted for the US population, the methods and key results were deemed generalizable to Canada.

### Ethics, equity, feasibility and acceptability considerations

Expanding RZV use to adults who are immunocompromised could address disparities in disease risk and access to vaccination. There is variability in public funding for HZ vaccination across provinces and territories, with only a few jurisdictions publicly funding RZV for immunocompromised adults as of late 2024, when NACI deliberated on this topic. Expanding existing programs to include immunocompromised populations or creating new programs for immunocompromised populations could promote equitable protection for individuals at high risk of HZ and its complications who face financial barriers.

Feasibility was deemed higher in provinces and territories with existing RZV programs and in jurisdictions that have other vaccination programs that specifically include immunocompromised populations. Recombinant zoster vaccine is an inactivated, refrigerator-stable, two-dose vaccine that can be concurrently administered with other vaccines and is thus well-suited for routine immunization programs. While there was limited evidence on acceptability among healthcare providers and the public, some surveys have reported coverage estimates indicating that nearly half of immunocompromised adults aged 50 and older had received at least one dose of an HZ vaccine ((31)). Internationally, RZV is recommended for individuals who are immunocompromised in several jurisdictions, including Australia, US, the European Union, New Zealand and Japan.

## Recommendations

The following recommendation is to inform RZV immunization programs for Canadian provinces and territories:

NACI recommends that individuals 18 years of age and older who are or will be immunocompromised should receive two doses of RZV to prevent HZ and its associated complications. ***(Strong NACI recommendation)***

The standard schedule is two doses administered two to six months apart; however, if needed, for individuals who will be at increased risk of HZ due to immunodeficiency or immunosuppression (for example, individuals who are about to start immunosuppressive therapy), the second dose can be administered at a minimum interval of at least four weeks after the first dose, as these individuals will benefit from completing the series before being immunosuppressed. To optimize immune response, the series should ideally be completed at least 14 days before the onset of immunosuppression.

The following list of immunocompromising conditions is intended to support the prioritization of individuals for vaccination but is not a comprehensive list of all immunosuppressive conditions or therapies. Since the degree of immunosuppression and associated risk of HZ can vary, clinical judgment and consultation with the patient’s healthcare provider are recommended.

• Primary immunodeficiencies affecting innate, humoral and T cell-mediated immunity

• Hematopoietic stem cell transplants (HSCT)

• Solid organ transplants (SOT)

• Hematological malignancies

• Solid tumour malignancies on immunosuppressive treatment

• HIV infection

• Chronic or ongoing immunosuppressive therapy:

ο Immunosuppressive chemotherapy

ο Immunosuppressive radiation therapy

ο Calcineurin inhibitors

ο Cytotoxic medications

ο Anti-metabolites

ο Immune effector cell therapies (e.g., CAR T cell therapy)

ο Biological response modifiers, targeted therapies and antibodies that target lymphocytes and immune pathways (e.g., anti-CD20, anti-TNF-α, JAK inhibitors, etc.)

ο Long-term, high-dose systemic corticosteroids (prednisone equivalent of ≥2 mg/kg/day, or 20 mg/day if weight of >10 kg for ≥14 days)

### Additional guidance

• There are no data on RZV use during pregnancy or breastfeeding, thus, precautions should be used in these situations. Ideally, vaccination should occur prior to pregnancy or deferred until after pregnancy. Recombinant zoster vaccine can be used in breastfeeding women and breastfeeding individuals if clinically indicated.

• Recombinant zoster vaccine can be concurrently administered with live and non-live vaccines.

• Individuals who have never been infected with VZV and have not received the varicella vaccine are not at risk of developing HZ; however, neither serologic testing nor confirmation of prior VZV exposure are required before administering RZV to eligible individuals.

• Individuals who have acquired immunity to VZV through vaccination rather than natural infection have a lower risk of HZ; however, RZV is still likely of benefit and should be offered if eligibility criteria are met.

• As RZV is not intended to prevent primary VZV infection, individuals known to be VZV-susceptible should be assessed according to current varicella vaccine guidelines in the Canadian Immunization Guide. It is important to consider that live vaccines, including varicella, may be contraindicated in some immunocompromised individuals.

## Conclusion

This updated recommendation from NACI reflects a growing body of evidence supporting the safe and effective use of the RZV in adults who are immunocompromised. Immunocompromised individuals face a significantly higher risk of HZ and its complications compared to the general population. The demonstrated efficacy, effectiveness, immunogenicity and safety of RZV in this population, along with international alignment, underscore the importance of including this group in routine immunization strategies. Expanding access to RZV for adults aged 18 years and older who are or will be immunocompromised is expected to reduce the burden of HZ and promote equity by minimizing financial barriers to vaccination. As provinces and territories consider implementation, this guidance provides a foundation for the development or expansion of publicly funded RZV programs that prioritize individuals at greatest risk. NACI will continue to monitor the evidence of vaccine efficacy, effectiveness, immunogenicity and safety of RZV in immunocompromised populations and other research priorities as outlined in the statement.
